# The Impact of Social Disparities on Microbiological Quality of Drinking Water Supply in Ugu District Municipality of Kwazulu-Natal Province, South Africa

**DOI:** 10.3390/ijerph16162972

**Published:** 2019-08-18

**Authors:** C. M. N. Khabo-Mmekoa, M. N. B. Momba

**Affiliations:** 1 Department of Biomedical Technology, Arcadia Campus, Tshwane University of Technology, 175 Nelson Mandela Avenue, Arcadia, Pretoria 0001, South Africa; 2 Department of Environmental, Water and Earth Sciences, Arcadia Campus, Tshwane University of Technology, 175 Nelson Mandela Avenue, Arcadia, Pretoria 0001, South Africa

**Keywords:** improved drinking water sources, water-borne disease, social disparity

## Abstract

This study was undertaken to highlight the social disparity between rural and urban areas in terms of housing patterns, provision of safe drinking water, access to sanitation facilities, education, employment rate and health-related to diarrhoeal episodes in Ugu District Municipality of KwaZulu-Natal Province of South Africa. To achieve this aim, a survey was conducted using a structured questionnaire. Drinking water samples were collected from the point of supply and the storage containers to assess the microbiological quality of drinking water in both rural and urban areas. Results of this study revealed prominent residential segregation between rural and urban communities, whereby the houses in the rural areas were generally constructed with corrugated iron sheets, or mud brick and mortar whereas conventional brick-and-mortar construction was used to build those in the urban areas. All of the urban households had flush toilets in their houses (100%), while 98.2% of the rural households were relying on pit latrines and 1.8% were reported to defecate in an open field. The District unemployment rate was at 58.1% in rural areas and none among the urban community. Results also showed that only 13.6% of the rural dwellers completed their secondary education compared to 70.4% of the urban areas. The diarrhoeal episodes were high in rural areas (34.1%) while none of these episodes was reported in urban areas. Great disparity in the water supply persists between rural and urban communities. For the former, the standpipes located outside their homes (90.9%) remain the sole mode of access to drinking water, while in the urban area, all households had pipes/taps inside their houses. Assessment of the drinking water quality revealed only the stored drinking water used by the rural community of Ugu District was contaminated. High prevalence of *E. coli* ranging from 63.3 % to 66.7% was recorded only in stored water after the sequencing of 16S rRNA genes. Species-specific PCR primers exposed the presence of enteropathogenic *Escherichia coli* at a rate ranging between 1.4% and 3.7% in this water Overall, this study has been able to highlight the disparity left by the legacy of racial segregation in the Ugu Municipality District. Therefore, the local government must intervene in educating homeowners on safe water storage practices.

## 1. Introduction

The advent of community water services was considered as one of the greatest public health advances of the 20th century, yet this has not been the case for rural or minority communities worldwide [1]. One of the major challenges faced by humanity in the 21st century is the lack of adequate provision of drinking water and sanitation coverage. The main aim of the Millennium Development Goals was to push back poverty, inequality, hunger, illness, and to reduce by half the proportion of people who had no sustainable access to safe drinking water in 1990, and therefore, to extend access to 88% of the global population [2]. Between 1990 and 2015, the number of people having access to improved drinking water sources rose to 2.6 billion [3]. However, this claim was not substantiated by the Joint Monitoring Program that showed a considerable change in the level of compliance with types of improved sources according to WHO water safety guidelines. [4]. The MDG target of 88% coverage for access to improved drinking water was met in 2010 [5]. While tremendous progress was achieved by 2015, 663 million people still drank from unprotected sources, and amongst these, 159 million solely depended on surface water [3]. Access to clean and safe drinking water was shown to be significantly high in urban areas, but unequal (slow) progress was made among marginalised and vulnerable groups. Reports have shown that 82% of the world’s population without improved drinking water sources lives in rural areas [5].

The inequalities between and within countries bring a large gap between the richest and the poorest [6]. This problem is experienced worldwide both in developed and developing countries. In the United States of America, for instance, the lack of access to clean and safe drinking water is still strongly linked to race for many communities in spite of a well-developed legal and regulatory framework governing the quality and provision of water at both the federal and state level [7]. MacDonald and co-authors [8] have shown that, in cities and towns of North Carolina, African American communities have been systematically denied access to municipal drinking water services. This inequality is also observed in low-income and people of colour throughout California where communities are exposed to unsafe drinking water [7]. However, the water supply infrastructure ranges from large systems serving millions of people to private wells for a single-family. The infrastructure provides piped water to the homes of over 99% of the US population. Despite such high levels of access provided, reports from several parts of the country still indicate disparities in access to piped and/or potable water [7,8]. 

The inequality trend experienced in the United States of America in terms of access to safe drinking water is similar to that of black and minority communities residing in rural and peri-urban areas of South Africa. The inequality in South Africa originated from the racial lines during the apartheid era. It denied equal access to education, employment, services, and resources. Black communities residing in rural areas and townships endured most of these inequalities. This was compounded by a lack of funding and sufficient experience of black local authorities in planning and designing water infrastructure for their communities [9]. While South Africa is one of the few countries that preserve the constitutional right for all of its citizens to be provided with an environment not harmful to their health or well-being [10], to date, the inequality in the provision of clean and safe drinking water still persists in some areas of the country. In almost all of the South African urban metropolitan areas, the infrastructure for water treatment and supply to consumers is of high quality compared to rural areas where it is poor or non-existent [11,12,13,14,15,16,17]. Urban communities are provided with safe drinking water that meets the South African National Standard [18] Drinking Water Specification. In contrast, rural communities rely on open water sources or collect water from a communal standpipe erected outside their houses where they have to collect water and store it until needed. The country is facing challenges in addressing the huge service backlogs of providing water and sanitation. In 2015, 3.64 million people in South Africa still had no access to an improved water supply [19]. 

In Ugu District Municipality of the KwaZulu-Natal Province, around 40% of the population still do not have access to potable water, which means that in 2010 about 263,000 Ugu District residents were living without access to safe drinking water [20]. Rural areas lack basic amenities, which include proper infrastructures such as roads, electricity supply, proper sanitation, and health facilities. A broad spectrum of bacteria is reported to compromise water quality with dire consequences for public health, particularly in smaller communities and developing countries where water is accessed from open sources [21]. The microbiological quality of drinking water is, therefore, a matter of concern to consumers, water suppliers, regulators, and public health authorities. Although the contamination of water with faecal bacteria is a common and persistent worldwide problem [22] the situation in rural and minority communities of developing countries remains perilous. This requires drinking water to be regularly monitored for faecal indicator bacteria [23]. Due to the difficulties to detect potential pathogenic bacteria (such as *Salmonella* spp., *Shigella* spp., and diarrhoeagenic *E. coli*), protozoan parasites (such as *Giardia lamblia* and *Cryptosporidium parvum)* and enteric viruses, concentrations of faecal bacteria including thermotolerant coliforms, enterococci and *E. coli*, are used as the primary indicators of faecal contamination [24]. 

The present study was therefore undertaken to establish the impact of social disparities on the quality of drinking water supplied to rural and urban communities by the Ugu District Municipality in KwaZulu-Natal, South Africa. To achieve the aim of the study, two main objectives were pursued. Firstly, a quantitative cross-sectional survey was used to ascertain the social discrimination in terms of housing, employment rate, and level of education, the physical infrastructure, and mode of access to municipal drinking water sources and the incidences of diarrhoeal diseases within both urban and rural areas of Ugu District Municipality. Secondly, the quality of municipal drinking water supplied to both rural and urban communities was assessed by tracking thermotolerant coliforms and *E. coli* from the point of treatment to the point of use.

## 2. Materials and Methods

### 2.1. Description of the Study Site and Population

**Figure 1:**
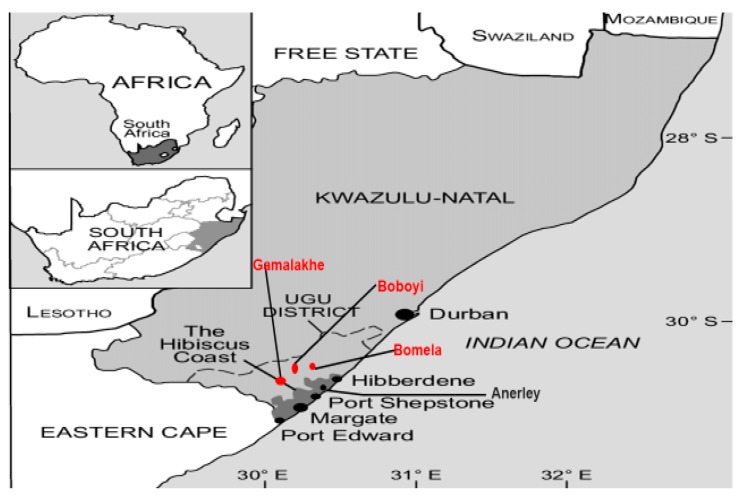
Map showing the study area at Ugu District Municipality, KwaZulu-Natal Province, South Africa.

The Ugu District Municipality is situated in KwaZulu-Natal Province on the border between the KwaZulu-Natal and Eastern Cape Provinces. The Ugu nodal area covers approximately 5866 km^2^ and has a population of approximately 700,000. The Ugu District Municipality was selected because of the high HIV prevalence rate (44%), high unemployment rate (30%), the backlog regarding the provision of water services to the population (70%), and the population distribution by race (89% black, 5% white, 3% Indians, 1% coloured and 2% other). While 16% of the population is located in the urban coastal strip, the balance of 84% resides in the rural areas, which are characterized by a low density and a dispersed settlement pattern. Approximately 50% of the population falls in the age group of 15 to 64 years [20]. Statistical data obtained from the HIV/AIDS clinics at the Murchison and Port Shepstone hospitals indicated that most of the patients originated from Anerley, Boboyi, Bomela, Gamalakhe, Hibberdene, Margate and Port Shepstone. The geographic position and drinking water sampling sites are depicted in Figure 1.

**Figure 2:**
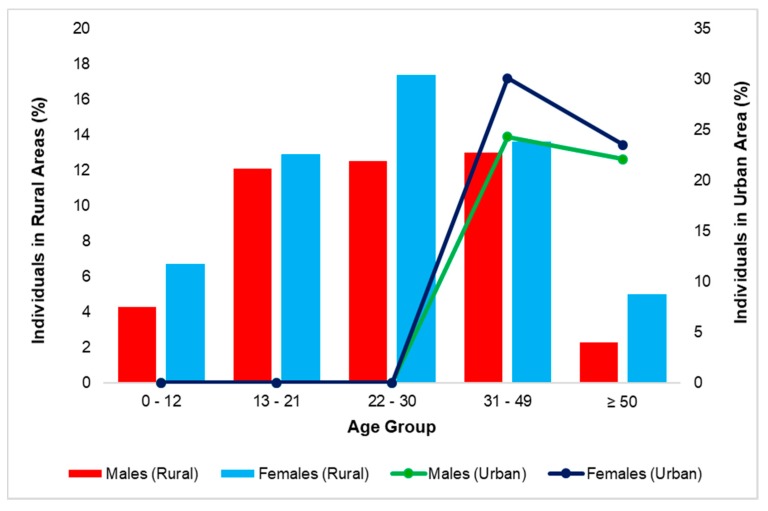
Population distribution of the selected areas at Ugu District Municipality during the study period.

The households in rural (220) and urban (108) areas, which were randomly selected during the study period had a population size of 1533 and 226, respectively. Amongst these populations, the most dominant group were females aged between 22 and 30 years (17.4%) and 31 to 49 years (29.6%) in rural and urban areas, respectively. Moreover, at the time of the survey, there were no children under the age of 12 years and no age group of between 13 to 30 years in the urban areas. Figure 2 provides the demographic information of the selected areas in Ugu District captured during the study period.

### 2.2. Scientific Ethics and Informed Consent

Prior to conducting the study, an Ethical Clearance was obtained from the Ethics Committees of the Tshwane University of Technology (TUT) and Ugu District Municipality. Informed consent to participate in the study was obtained from the owners of houses and the plant manager. A questionnaire was developed and a clear justification of the aim and objectives of the study was provided to the study participants. 

### 2.3. Study Survey on Social Discrimination

A quantitative cross-sectional survey using a structured open-ended questionnaire was conceptualised in English. For the participants who could not speak and understand English, the questions were translated and administrated in Zulu (the native language of the black community in the KwaZulu-Natal Province). The survey was based on the patterns of housing in rural and urban areas, the number of people occupying this housing, employment rate, the level of education, the physical infrastructure (e.g., treatment facilities, transmission, and storage) and the mode of access to municipal drinking water sources as well as incidences of diarrhoeal diseases in the target communities. Interviews were done face to face in respondents’ homes. In the event that the relevant information was not provided by the participants, statistics reports published between 2002 and 2015 were also sourced from Statistics South Africa, although the study was conducted between January 2008 and November 2009, and also in April 2015. These data were crucial to establishing the effort provided by the country to eradicate social disparities between rural and urban area communities.

### 2.4. Collection of Drinking Water Samples

A total of 1867 drinking water samples were collected over a period of 12 months between January 2008 and November 2009 and in April 2015 in rural and urban areas. As the year 2015 matched to the end of the MDG Target 7c, which aimed to reduce by half the proportion of people without access to safe drinking water, it was imperative for the investigators to establish whether a change in the mode of access to drinking water and the quality had occurred during this period. For the communities with taps, these were sterilised with 70% ethyl alcohol and the water was allowed to flow for approximately one minute prior to collection. The samples were collected in 1 L sterile bottles containing 120 mg of sodium thiosulphate (Na_2_S_2_O_3_) to neutralise the residual chlorine in the water [25]. Thereafter all drinking water samples were transported on ice in a cooler box to the Boboyi Water Treatment Plant laboratory where the initial analyses were performed within 4 h to 6 h of collection. 

### 2.5. Analysis of Drinking Water Quality

#### 2.5.1. Physicochemical Characteristics of Drinking Water Samples

The residual chlorine concentration, turbidity level, temperature and the pH of the drinking water samples were determined on-site according to the Standard Methods for the Examination of Water and Wastewater [26]. The physicochemical parameters were compared against the standards set by SANS 241 [17] and the Water Quality Guidelines for Domestic Use [27]. 

#### 2.5.2. Microbiological Characteristics of Drinking Water Samples

##### Detection and Enumeration of Culturable Thermotolerant Coliforms and *E. coli*

The membrane filtration techniques were used for the detection and enumeration of the target coliform bacteria according to the standard methods [26] using a lactose-based agar [m-FC: membrane faecal coliform (BioLab)] for thermotolerant coliforms (faecal coliform) and Chromocult^®^ coliform agar [CCA (Merck)] for total coliforms and *E. coli.* The agar plates were prepared according to the manufacturer’s instructions. Incubation of the agar plates for thermotolerant coliforms was performed at 44.5 °C and for total coliforms and *E. coli* at 36 ± 1 °C for 18–24 h. Plates were always in triplicate for each type of organism. The abundances of coliforms were reported as colony-forming-unit (CFU) per 100 mL of drinking water. 

Individual colonies were randomly selected based on their size, shape, and colour and inoculated in 2 mL Nutrient broth, incubated overnight, preserved with 20% glycerol and transported on ice packs to Microbiological Laboratory of the Water Research Group at the Tshwane University of Technology, South Africa, for further analysis. In the laboratory, the individual bacterial colonies from glycerol were streaked onto the corresponding selective media by the streak plate method and incubated for 24 h at 44.5 °C for thermotolerant coliform and at 36 ± 1 °C for total coliforms and *E. coli*. The colonies were purified further using the same method at least three times with nutrient agar (BioLab). A series of microbiological analyses including catalase and oxidase production and biochemical tests (using the API 20E) were used to confirm suspect *E. coli* [28,29,30]. All oxidase negative colonies were transferred onto nutrient agar slants, incubated at 36 ± 1 °C for 24 h and stored at 4 °C for further use. 

##### Molecular Identification of the Isolates

Extraction of total genomic DNA—For the molecular study, a total of 287 oxidase-negative isolates were used. Individual isolates were grown in nutrient broth, followed by incubation at 36 ± 1 °C for 24 h. The inoculated broths (1 mL) were centrifuged at 16,000 *g* for 5 min. The pellets were washed twice with sterile molecular grade water. Total genomic DNA was extracted from the bacterial pellets using the DNeasy^®^ DNA purification kit (QIAGEN) and ZR Fungal/Bacterial DNA MiniPrep^TM^ kit (ZYMO Research, Irvine, CA, USA), in accordance with the manufacturer’s instructions. The quality and quantity of the isolated nucleic acids were determined using the NanoDrop^TM^ 2000 spectrophotometer (Thermo Scientific, Johannesburg, South Africa) and by agarose electrophoresis (Bio-Rad) on a 1.5% agarose gel electrophoresed at 100 V.

Restriction Analysis of PCR Amplicons and Identification of the Virulence-Associated Genes

In order to select representative isolates for sequencing, all PCR amplicons were subjected to restriction analysis. For this purpose, 10 μL of the 16S rRNA amplicons were digested with Taq1 and Cs6pI (Fermentas) according to the manufacturer’s instructions. The restriction digest fragments were separated using conventional electrophoresis on a 1.5% (*w*/*v*) agarose gel stained with ethidium bromide, followed by visualisation under ultraviolet light. The HyperLadder™ 1 kb, 100 lanes (Bioline Products, Pretoria, South Africa) was included as a size marker. The results were captured using a gel documentation system (Syngene, Cambridge, UK). The restriction patterns were determined manually and for every five similar profiles, one isolate was selected for sequencing. A total of 287 isolates that tested positive for the *E. coli* bacterium were amplified using species-specific primers (Table 1) to test for the virulence gene. 

All 287 confirmed *E. coli* samples were selected to be tested for the virulence gene. Species-specific PCR primers were used to amplify the eae, stx, ipaH, and aggR genes (Table 1). The PCR reaction mixtures contained 12.5 μL of DreamTaq Master mix (2×) (Fermentas, 140 St. Leon-Rot, Germany), 0.5 μL of each primer), 8.5 μL of nuclease-free water (Fermentas, 140 St. Leon-Rot, Germany) and 5 μL of template DNA. A negative control with no DNA template and a positive control with DNA of *E. coli* ATCC 25922 (Quantum Biotechnologies, Cape Town, South Africa) were included in all the PCR experiments. The PCR reaction mixtures were placed in an MJ Mini™ thermal cycler (Bio-Rad Laboratories, Johannesburg, South Africa) to amplify the DNA. This was done with the following thermal cycling conditions: pre-denaturation for 10 min followed by 35 amplification cycles of denaturation at 94 °C for 30 s, annealing of primers with template DNA at 55 °C for 30 s, and primer extension at 72 °C for 30 s. This was followed by a final extension at 72 °C for 7 min. The PCR amplicons were separated on a 1% (*w*/*v*) agarose gel stained with ethidium bromide electrophoresed at 100 V and visualised under ultraviolet light. The FastRuler™ Low Range DNA ladder (Fermentas, 140 St. Leon-Rot, Germany) was included in all the gels as a size marker. The results were captured using a gel documentation system (Syngene, Cambridge, UK). The isolates were then sent to Inqaba Biotechnical Industries (Pty) Ltd. (www.inqababiotec.co.za), Pretoria, South Africa for confirmation and Sanger sequencing of the 16S rRNA gene.

## 3. Results

### 3.1. Social Discrimination of the Ugu District during the Study Period

#### Disparities in Housing Patterns

**Figure 3:**
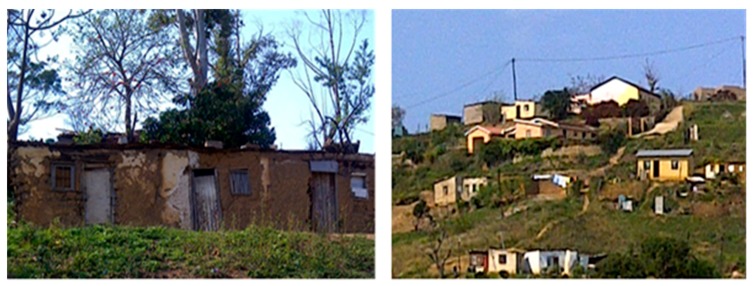
Types of houses in the rural settlement.

**Figure 4:**
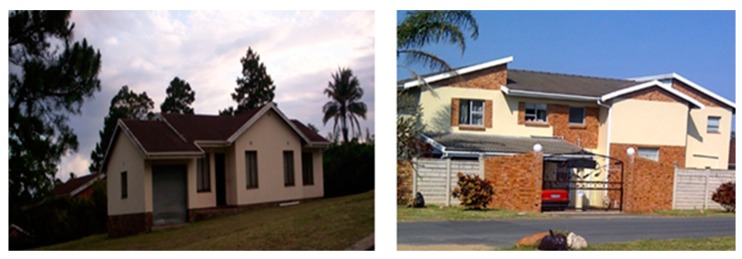
Types of houses in the urban settlement.

**Figure 5:**
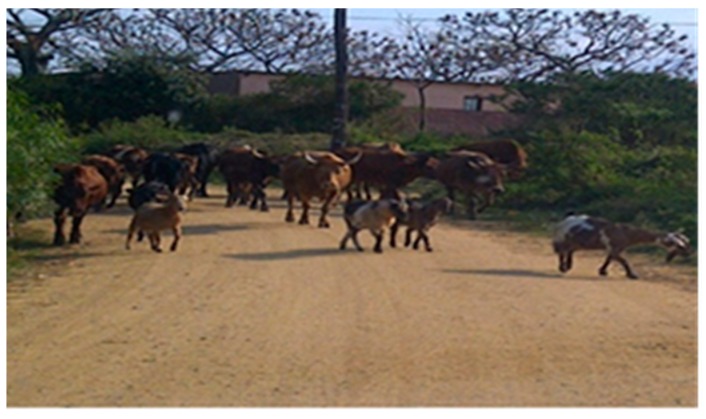
Livestock found in rural areas.

The demographic profile of Ugu District Municipality assisted in conducting a proper socio-economic analysis of a region that showed the differences in the patterns of houses provided for the rural and urban residents. Results from this study revealed prominent residential segregation between rural and urban communities as shown in Figure 3 and Figure 4, respectively. The houses of Boboyi and Bomela were generally constructed with corrugated iron sheets, or mud-brick and mortar, whereas in Gamalakhe houses were built mainly with mud bricks and mortar. The residents of these housings were 100% black. The observation made during the study period indicated that the geographic location of dispersed houses made it difficult for the sustainability of planning and access to basic amenities. The physical environment of the rural houses permitted sharing of their land with their livestock (Figure 5). In urban areas, conventional brick-and-mortar construction was used to build houses. There was no overcrowding observed as the houses were well planned and designed with a solid structure. 

### 3.2. Disparities in Water Supply and Sanitation Facilities in the Target Study Area

A greater disparity in water supply and sanitation facilities was observed during the period of the study between rural and urban areas. Table 2 outlines the results of the disparities in water and sanitation in the target rural and urban areas. The results showed that none of the rural households had water pipes inside their houses while 100% of the urban households had access to piped water inside their houses. The rural dwellers were found to have the tap either within the yard (1 m–2.5 m) (9.1%) or collecting water from the communal tap ≥200 m away from their homes (90.9%). The sanitation facilities were also assessed and there was a great disparity between the rural and urban areas. All of the urban households had flush toilets in their houses (100%) while 98.2% of the rural households were relying on pit latrines and 1.8% were reported to defecate in an open field. The diarrhoeal episodes were high in rural areas (34.1%); moreover, none of the diarrhoeal episodes were reported in urban areas. 

### 3.3. Disparities of Employment Rate and Level of Education in the Target Study Area

Table 3 displays the disparities of employment and level of education between rural and urban areas of the Ugu district. During the study period, the district unemployment rate was moderately high (58.1%) in rural areas as compared to the (57.1%) South African national jobless rate (20). In contrast, none of the urban community members was unemployed. Moreover, the results of the survey revealed greater disparities in the level of education between rural and urban areas. The results showed that only 13.6% of the rural dwellers completed their secondary education compared to 70.4% of the urban areas. Furthermore, the survey revealed that the majority of the rural dwellers dropped-out of school (59.8%) while none of the urban dwellers dropped out of school.

### 3.4. Disparities in Drinking Water Infrastructure and Mode of Access to Municipal Drinking Water 

**Figure 6:**
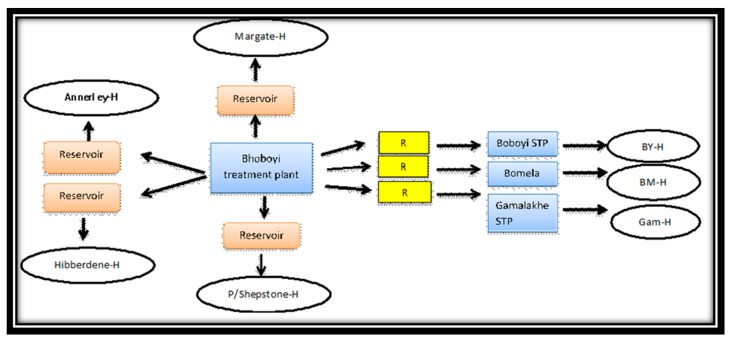
Mode of access to municipal drinking water supply in target urban and rural areas (H: house; R: reservoir; STP: Standpipe; BY-H: Boboyi house; BM-H: Bomela house; Gam-H: Gamalakhe house).

As can be seen in Figure 6, both rural and urban communities of Ugu District Municipality receive their drinking water from the Boboyi Water Purification Plant. This plant abstracts its intake water from the Umzimkhulu River and produces drinking water using conventional methods (coagulation, flocculation, sedimentation, rapid sand filtration, and chlorination). 

**Figure 7:**
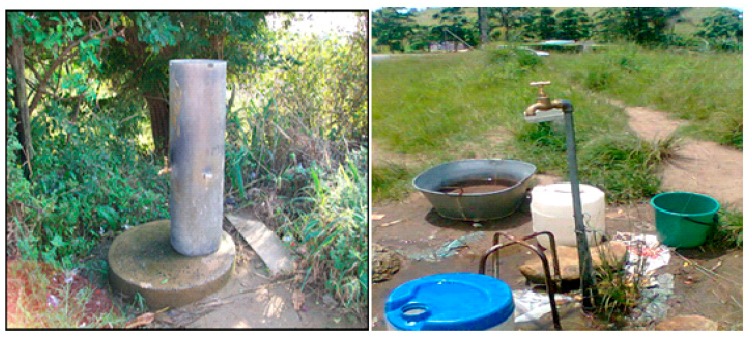
Different rural communal standpipes in Boboyi, Bomela and Gamalakhe supplying drinking water to households.

**Figure 8:**
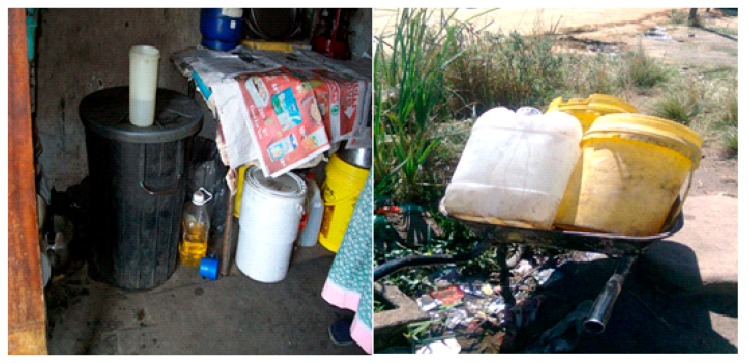
Containers used for the collection of drinking water from the communal standpipes and for the storage of water in the Boboyi, Bomela and Gamalakhe dwellings.

In rural areas, drinking water is supplied from the treatment plant to a reservoir, and it is delivered to the communal standpipes situated outside houses (Figure 7) where it is collected and stored in buckets (Figure 8). Based on the study survey conducted between 2008 and 2009, there were a total of 27 standpipes that supplied drinking water to 220 houses visited during this period.

**Figure 9:**
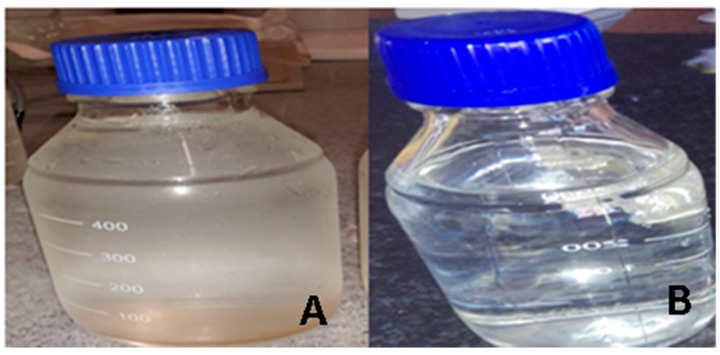
Quality of water produced in the rural area (**A**) and urban areas (**B**) of Ugu District Municipality.

In urban areas, the water follows the same procedure to the reservoir, but from the reservoir, it is delivered directly to the taps inside dwellings (Figure 6). Margate, Port Shepstone, Annelin, and Hibberdene are provided with indoor plumbing and tap(s) are fitted inside each house. All of the residences (108) surveyed during the study period reported that they had a kitchen sink with hot and cold piped water. Visual observation of drinking water quality (Figure 9) showed that the communal standpipes produced turbid water compared to urban household taps that supplied clear water. 

### 3.5. Water Quality Analysis

#### 3.5.1. Physicochemical Characteristics of Water Samples from Rural and Urban Areas of Ugu District

Results of the study revealed that the turbidity values for drinking water samples collected from both urban and rural areas by far exceeded the recommended South African National Standard SANS 241 limits. In rural areas, the mean turbidity value ranged between 2.4 and 3.3 NTU for standpipes and between 2.8 and 4.7 NTU for container-stored water. The highest turbidity levels in both standpipe and container-stored drinking water were found in samples collected from Boboyi, following by Bomela and Gamalakhe (Table 4). The results for temperature was found to be within the acceptable limits set by WHO and SANS in both rural and urban areas. Although the turbidity of the water samples from the urban areas was found to be less than that of the rural areas, it was, however, not within the acceptable limits set by both WHO and SANS. The turbidity ranged between 0.8 NTU at the point of treatment and 1.9 NTU in homes. The temperature values, however, were found to be within the acceptable limits. 

#### 3.5.2. General Microbiological Quality of Drinking Water Supply in the Rural and Urban Area of Ugu Municipality 

Two hundred eighty-seven (287) samples were analysed for the presence of presumptive enteropathogenic bacteria using culture-based methods. As the results obtained during the sampling regime of 2008 and 2009 did not remarkably vary in terms of bacterial counts compared to those obtained in 2015, the geometric mean concentrations of presumptive coliform bacteria were combined (Table 5). Of all the coliforms bacteria enumerated from storage containers of rural areas, thermo-tolerant coliform counts of 1.477–1.653 log_10_ CFU/100 mL were found to be more prevalent than the faecal coliform and the *E. coli* counts. These counts ranged from 1.301 to 1.544 log10 CFU/100 mL and from 1.230 to 1.255 log10 CFU/100 mL in all three rural areas, respectively. The results further highlighted disparities between rural and urban areas as none of these targeted coliforms were detected in water samples from the urban area. 

#### 3.5.3. The Prevalence of Pathogenic *E. coli* Detected in Drinking Water Samples from Rural and Urban Areas of Ugu District after Sequencing of 16S rRNA Genes 

Table 6 illustrates the results for the prevalence of pathogenic *E. coli* detected from water in both rural and urban areas of Ugu district during the study period. The results showed the greater disparity between drinking water quality supplied to rural and that consumed in urban areas. Higher prevalence was of *E. coli* was found in Bomela (66.7%) followed by Boboyi (65.4%) and Gamalakhe (63.3%). In contrast, none of the target pathogenic bacteria were detected in water samples from urban areas. Of all three rural areas, Gamalakhe was found to have the lowest prevalence of all target pathogenic bacteria. 

The specific virulence genes for EHEC, EPEC, EIEC, and EAEC were identified using species-specific PCR primer and the results are shown in Table 7. The samples that tested positive for the eae gene of EPEC were from the rural storage containers of Boboyi (3.7%), Bomela (2.7%) and Gamalakhe (1.4%). However, only 1.5% of these pathogenic bacteria were detected from standpipe water samples collected from Boboyi Village (Table 7). None of the pathogenic *E. coli* was detected in drinking water collected from urban dwellings.

## 4. Discussion 

### 4.1. Social Disparities in Housing, Education Level, Employment Rate, and Health

Given the inheritance of inequalities and injustices of the past, the provision of housing has remained a major challenge in South Africa. Regardless of the positive developments made by the South African government since 1994, the disparities between the underprivileged and the affluent in the home still continue unabated [35]. As a result, the underprivileged population encounters challenges to access housing due to pecuniary difficulties, leaving the affluent to continue enjoying the acquisition of houses. In this study, such disparities were observed during the survey (Figure 3 and Figure 4). The evidence showed that the houses in rural communities were of poor quality and most of them were built from mud bricks, while those of the urban areas were of good quality built using face brick. This situation may affect negatively the health of communities in rural areas. Reports in Europe and the United States have pointed out the higher number of deaths in rural areas due to lack of adequate ventilation of the apartments and lack of social support for the elderly people trapped in them. In most cases, mostly affected households are low-income earners as it is mostly likely that they can afford air conditioning such as fans. Moreover, low-income areas are less likely to be in housing surrounded by trees or shrubs, which can provide a cooling effect and encourage recreation [36] as they live in contact with livestock (Figure 5), which might feed on the vegetation. 

At a social level, it has been highlighted that lack of proper housing is strongly associated with health; those who live in good quality houses are usually in better health, while those residing in poor quality houses are usually exposed to various diseases such as waterborne diseases. Barker et al., [37], reported that household density and overcrowding are related to communicable diseases such as tuberculosis and meningococcal disease, in addition to diarrhoeal diseases. Household density and overcrowding usually happens in rural areas where the majority of people are unemployed. The results of this study confirmed the report made by Barker et al., [37] as it was recorded that none of the urban dwellers surveyed had diarrhoea during the study period, while 34.1% of the rural dwellers surveyed in this study had diarrhoea (Table 2). 

In many rural societies, a lack of access to education and limited opportunities to increase and improve one’s skills set inhibit social mobility [38]. Low levels of education and few skills have been shown to result in individuals from poor rural working as subsistence farmers or in insecure, informal employment, which perpetuates the state of rural poverty. These conditions were also observed in this study, whereby the majority of the rural dwellers had dropped out of school (59.8%). Consequently, these people are working only as security officers (13.6%) and most of them were not employed (58.1%) because of a lack of skills. Moreover, inequality between urban and rural areas, and where rural poverty is most prevalent, is in countries where the adult population has the lowest level of education. This was also found in the Sahel countries of Burkina Faso, Mali, and Niger where regional inequality is 33%, 19.4%, and 21.3%, respectively. In each of these countries, more than 74% of adults have no education. Overall, in most of Africa, those living in rural areas experience more poverty and less access to health care and education [39]. 

Education helps to attain the quality of life. Results of this study showed that the urban settlers had a better educational background than the rural settlers. This was evident in the types of their dwelling place as well as the types of work the residents have. This clearly shows the influence of education on the employment rate. Numerous studies have shown the increasing disparities between the people of colour and the whites. These were linked to the physical and social environments based on the traditional domains of the planning and civil engineering, and residents being unable to access basic amenities, and have led to adverse health [40,41]. It has been reported that people with high incomes tend to suffer less from ill-health than people with lower incomes. Furthermore, the death rates among children from the former category are low and most often occur after the age of 60. This is quite different in communities with low income (defined by mud houses, broken windows with no chairs in some houses and no electricity, in this study); the death rates are higher, diarrhoeal diseases and HIV/AIDS are prominent and account for more deaths in females than in males [42]. 

### 4.2. Disparities in Sanitation, Water Supply, and Water Quality 

Suitability of water supply principally points to supplying adequate and quality water for the wellbeing of human health. In addition, sustainable access to basic sanitation, improved and safe drinking water was one of the Millennium Development Goals (MDGs) which was supposed to have been met by the year 2015 [43]. As stated above, in spite of the progress made in halving by 2015 the proportion of the population without sustainable access to safe drinking water and basic sanitation, the challenges facing many countries, especially in developing countries in these sectors, still remain overwhelming. The poor continue to be marginalised from most of the improvements that have been documented by WHO/UNICEF [43]. This study assessed the disparities in sanitation, water supply and water quality of the Ugu District in KwaZulu-Natal, South Africa. The results showed that disparities still exist between rural and urban areas in terms of sanitation as well as in access to improved water supply infrastructure and safe drinking water (Table 2) in Ugu District. 

Even though both rural and urban communities of the Ugu District were shown to be receiving safe drinking water from the same treatment plant, water was distributed to different reservoirs (Figure 6). Moreover, the residents in the urban areas were privileged to get the water from the taps installed in their houses, while the rural communities did not have the same privileges as they drew the water from the standpipes and stored it in household containers. The direct access to safe drinking water in urban areas provided a better level of service to the user as more quantities of water from the taps could be collected and used to fulfil the health and hygiene requirements of the householders. 

It is well known that water that is meant for human consumption should be free of faecal coliform bacteria in general and *E. coli* in particular. Nonetheless, results revealed the presence of this group of coliform bacteria in drinking water supplied through standpipes. This mode of access to drinking water in rural areas may jeopardise the health of the rural community. During the study period, it was observed that standpipes were located in the yards (Figure 7), where animal faecal matters were often found in stagnant water, which might infiltrate through broken water supply pipes within the yards. This is in-line with the findings of the study conducted by PieTrucha-urbaniK and co-workers [44], which highlighted that some of the problems arising from the operation of water supply systems are breaks in water supply which influence water quality when the standard requirements are not met. Moreover, the survival and growth of microorganisms in the water is dependent on several factors that include the temperature of the water, the turbidity and the level of chlorine, the pH, and other sources for their nutrients. Results of this study showed that the chlorine concentrations ranging between 0.13 and 0.08 in standpipe water could not inhibit the growth of the coliforms. High turbidity (2.4–3.3 NTU) might also affect the efficiency of free chlorine residual in inhibiting bacterial growth in standpipe water. Previous studies have repeatedly reported strong correlations between the level of turbidity and microbial contamination of treated water [8,13].

Another disadvantage is that the rural settlers had to walk a distance of about 200 m from their houses to collect the water that would be used to cover drinking, cooking, washing and in this regard, less than 20 L of water was used, thus limiting the householders to use the water as they wished. With the water sources not being located on the premises, members of the households need to spend time and energy to collect the water. The burden of water collection was left mainly to children or women in the houses. Women are forced to carry heavy buckets on their heads, which causes fatigue that not only harms their well-being but also affects productivity and reduces energy and time for economic opportunities [45]. In addition to the burden of water collection, women and girls are faced by the risk of sexual harassment when fetching the water from any available water source, which may be far away from their dwellings [46]. Additionally, the lack of access to clean and safe drinking water exposes rural communities to unhygienic conditions, which pose a huge health risk to the vulnerable population. There is often overcrowding, insufficient use of water for basic personal and domestic hygiene, poor waste disposal; all these contribute to the risk of outbreaks of waterborne diseases. Disparities in access to safe public drinking water are gradually being recognised as a significant contributing factor to health inequalities and environmental injustice for vulnerable communities. Reports have shown that poor drinking water quality or unsafe municipal water, denial of access to municipal water, contaminated home or well water, and even lack of indoor plumbing remain all the contributing factors [41,47,48,49]. 

Several studies have also demonstrated that the microbiological quality of drinking water deteriorates from source to point-of-use based on the hygiene practices [50,51,52]. Moreover, it is also documented that lack of proper hygiene practices, such as cleaning of drinking water storage vessels and dipping utensils used to remove drinking water from storage vessels, and washing of hands, as well as exposure of drinking water stored in open-top containers to dust and fomites contribute to poor microbiological quality of drinking water [51,53]. In this study, it was found that the water samples of the rural areas had an unacceptable level of contamination in terms of all the target pathogenic bacteria (*E. coli* and thermotolerant faecal coliform), while none of these organisms were detected in water samples from an urban area (Table 5). However, the water source (treatment plant) was the same. These results, therefore, support the findings of other researchers, which state that the microbiological quality of water deteriorates from the source to homes in storage containers [12,50,51,52,54]. Consumption of water, which is microbiologically contaminated, has been previously associated with increased rates of negative health outcomes such as diarrhoea [55,56,57]. This could explain a higher incidence of diarrhoea reported in the rural areas (Table 2) in this study. Moreover, poor microbiological quality of the water in storage containers in rural areas can be attributed to the presence of biofilm inside the storage containers. It is well documented that microorganisms require a surface with nutrients and flow of water to produce biofilm, which leads to an increase in the concentration of bacteria and turbidity of stored water [58,59,60]. The average mean turbidity of water from storage containers in rural areas was above the recommended levels in [61,62] (Table 4). High turbidity is typically associated with increased biofilm formation because of more particles being available to serve for attachment and nutritional purposes [60]. 

In South Africa, the communities reported to have the lowest rates of access to safe water and improved sanitation are in some of the poorest provinces of South Africa, which are Eastern Cape, KwaZulu-Natal and Limpopo. The majority of the population proportion in these provinces consists of rural communities of which most of them rely on unsafe drinking water. This matter is of greater disquiet, given the fact that safe drinking water is a human right and primary necessity for human being. It is well known that most of developing countries have a major challenge of finances not only to supply safe drinking water to their people. Based on the results of this study, there is an emergency call for policymakers to introduce inexpensive water treatment technologies capable to deliver safe drinking water to rural households. A well-designed sociodemographic profile should be readily available to provide vital information that could be used by the policymakers to allocate effective and sufficient funds that will improve service delivery, thereby improving the quality of many lives, especially in rural areas. Moreover, there is still a dire need for education on drinking water quality management and hygiene practices at household level in rural areas in general and in Ugu District Municipality in particular for the prevention of diarrhoeal diseases due to the consumption of contaminated drinking water. 

## 5. Conclusions and Recommendation

This study investigated the social disparities (housing, education level, and employment) and the disparities in sanitation, water supply and water quality in rural and urban areas of Ugu District. The results revealed major disparities in all the parameters between rural and urban areas. The same disparity was also evident in 2015 as access to drinking water and the water quality remained unchanged. The findings of this study demonstrate the need for the government to invest in the basic infrastructure for housing in rural communities to improve the lives of people living in rural areas. Water infrastructure for people living in rural areas must be one of the priorities for the eradication of waterborne diarrhoeal diseases such as malnutrition and dehydration. 

Furthermore, the findings of this study show that there is still a dire need for awareness/education on managing proper hygienic practices and protecting the water provided to rural communities (protection of collected water in homes). It is recommended that a well-designed sociodemographic profile should be readily available to provide vital information that could be used by the policymakers to allocate effective and sufficient funds that will improve service delivery, thereby improving the quality of many lives, especially in rural areas. Moreover, the findings of this study can be used in policymaking decisions to grant effective and sufficient funds for improving service delivery and consequently the quality of life. 

## Figures and Tables

**Table 1:** Species-specific primers used for the identification of diarrhoeagenic *E. coli*.

*E. coli* Strain	Designation	Sequence (5′ to 3′)	Target Gene	Product Size (bp)	Reference
**EPEC**	SK1	CCCGAATTCGGCACAAGCATAAGC	Eae	881	[31]
	SK2	CCCGGATCCGTCTCGCCAGTATTCG			
**EHEC**	VTcom-u	GAGCGAAATAATTTATATGTG	Stx	518	[32]
	VTcom-d	TGATGATGGCAATTCAGTAT			
**EIEC**	ipaIII	GTTCCTTGACCGCCTTTCCGATACCGTC	ipaH	619	[33]
	ipaIV	GCCGGTCAGCCACCCTCTGAGAGTAC			
**EAEC**	aggRks1	GTATACACAAAAGAAGGAAGC	aggR	254	[34]
	aggRks2	ACAGAATCGTCAGCATCAGC			

**Table 2:** An overview of water supply, sanitation facilities and episodes of diarrhoea in the target study areas.

Socio-Demographic Characteristics		Rural Areas		Urban Areas	
		Frequency (n = 220)	Percentage (%)	Frequency (n = 108)	Percentage (%)
**Water supply**	Inside houses	0	0	108	100
	Within the yard (1 m–2.5 m)	20	9.1	0	0
	Communal tap (≥ 200 m away)	200	90.9	0	0
	No access	0	0	0	0
**Sanitation facilities**	Flush toilets	0	0	108	100
	Pit latrines	216	98.2	0	0
	Open defecation	4	1.8	0	0
**Diarrhoeal episodes**	Yes	75	34.1	0	0
	No	145	65.9	108	0

During the interviews, participants who reported to have had loose/watery stools on more than three occasion per day were classified as having diarrhoea.

**Table 3:** An overview of the disparities in employment and education level in the Ugu District Municipality.

Socio-Demographic Characteristics		Rural Areas		Urban Areas	
		Frequency (n = 1533)	Percentage (%)	Frequency (n = 226)	Percentage (%)
**Employment rate**	Unemployed	890	58.1	0	0
	Domestic	177	11.5	0	0
	Security job	209	13.6	0	0
	Professional	189	12.3	126	55.8
	Retired/pensioners	68	4.5	100	44.2
**Education level**	Primary	130	8.5	0	0
	Completed secondary school	209	13.6	159	70.4
	Tertiary education	278	18.1	67	29.6
	Drop-out	916	59.8	0	0

**Table 4:** Mean turbidity, pH, temperature, and residual chlorine of water samples from the study area.

**Rural Area**												
**Parameter**	**Point of Treatment**			**Reservoir**			**Standpipe**			**Container-Stored Water**		
	**BY**	**BM**	**Gam**	**BY**	**BM**	**Gam**	**BY**	**BM**	**Gam**	**BY**	**BM**	**Gam**
**Turbidity (NTU)**	0.8	0.8	0.8	2.5	1.9	2.4	3.3	2.4	2.9	4.7	3.9	2.8
**Temperature (°C)**	26.4	26.4	26.4	25	25.6	25.3	25.4	24.8	25	23.2	22.7	22.6
**pH**	6.4	6.4	6.4	6.8	6.5	7.0	7.5	6.8	7.3	7.5	6.8	7.3
**Residual chlorine (mg/L)**	0.36	0.34	0.38	0.45	0.77	0.08	0.09	0.13	0.57	0.77	0.86	0.90
**Urban Area**												
**Parameter**	**Point of Treatment**				**Reservoir**				**Tap Water in Dwellings**			
	**ANN**	**HIB**	**MG**	**PS**	**ANN**	**HIB**	**MG**	**PS**	**ANN**	**HIB**	**MG**	**PS**
**Turbidity (NTU)**	0.8	0.8	0.8	0.8	1.5	1.9	1.6	1.5	1.5	1.8	1.6	1.5
**Temperature (°C)**	26.4	26.4	26.4	26.4	24.4	24.6	24.5	24.0	23	23.2	23.4	22.7
**pH**	6.4	6.4	6.4	6.8	6.7	6.7	6.8	6.6	6.8	6.8	6.9	6.6
**Residual chlorine (mg/L)**	0.14	0.36	0.1	0.36	0.33	0.32	0.38	0.41	0.31	0.40	0.37	0.81

BY: Boboyi; BM: Bomela; Gam: Gamalakhe; ANN: Annelin; HIB: Hibberdene; MG: Margate; PS: Port Shepstone.

**Table 5:** Geometric mean concentration (log10 CFU/100 mL) of presumptive *E. coli* and thermotolerant coliforms in Ugu District (2008–2009 and 2015).

**Rural Areas**												
	**Point of Treatment**			**Reservoir**			**Standpipe**			**Stored Water**		
	**Thermo-Tolerant Coliforms**	**Faecal Coliforms**	***E. coli***	**Thermo-Tolerant Coliforms**	**Faecal Coliforms**	***E. coli***	**Thermo-Tolerant Coliforms**	**Faecal Coliforms**	***E. coli***	**Thermo-Tolerant Coliforms**	**Faecal Coliforms**	***E. coli***
Boboyi	NG	NG	NG	NG	NG	NG	1.079	0.954	0.903	1.653	1.544	1.255
Bomela	NG	NG	NG	NG	NG	NG	0.954	0.778	0.602	1.477	1.301	1.176
Gamalakhe	NG	NG	NG	NG	NG	NG	0.602	0.477	0.301	1.556	1.342	1.230
**Urban Areas**									
	**Point of Treatment**			**Reservoir**			**Tap Water in Dwellings**		
	**Thermo-Tolerant Coliform**	**Faecal Coliforms**	***E. coli***	**Thermos-Tolerant Coliforms**	**Faecal Coliforms**	***E. coli***	**Thermo-Tolerant Coliforms**	**Faecal Coliforms**	***E. coli***
Annelin	NG	NG	NG	NG	NG	NG	NG	NG	NG
Hibberdene	NG	NG	NG	NG	NG	NG	NG	NG	NG
Margate	NG	NG	NG	NG	NG	NG	NG	NG	NG
Port Shepstone	NG	NG	NG	NG	NG	NG	NG	NG	NG

NG: No growth.

**Table 6:** Prevalence rates of *E. coli* detected in drinking water samples after sequencing of 16S rRNA genes (N = 287 selected samples, between January 2008, November 2009 and April 2015).

Organisms Detected	Rural Area (Storage Containers)			Urban Area (In-House Taps)			
	By	Bm	Gam	Ps	Mg	Ann	Hib
	N = 107	N 90	N = 90				
***E. coli***	65.4 % (70)	66.7 % (60)	63.3 % (57)	0 % (0)	0 % (0)	0 % (0)	0 % (0)

BY: Boboyi; BM: Bomela; Gam: Gamalakhe; ANN: Annelin; HIB: Hibberdene; MG: Margate; PS: Port Shepstone.

**Table 7:** The distribution of pathogenic strains of *E. coli* identified in drinking water collected from January 2008 to November 2009, and April 2015 from the Ugu District Municipality, KwaZulu-Natal Province, South Africa (N = 287).

Rural Areas	Point of Treatment	Reservoir	Standpipe				Stored Water			
	Pathogenic *E. coli*	Pathogenic *E. coli*	EHEC	EPEC	EIEC	EAEC	EHEC	EPEC	EIEC	EAEC
Boboyi	ND	ND	ND	1 (1.5%)	ND	ND	ND	2 (3.7%)	ND	ND
Bomela	ND	ND	ND	ND	ND	ND	ND	1 (2.7%)	ND	ND
Gamalakhe	ND	ND	ND	ND	ND	ND	ND	1 (1.4%)	ND	ND
**Urban Area**										
Margate	ND	ND	ND	ND	ND	ND	ND	ND	ND	ND
Annelin	ND	ND	ND	ND	ND	ND	ND	ND	ND	ND
Hibberdene	ND	ND	ND	ND	ND	ND	ND	ND	ND	ND
Port Shepstone	ND	ND	ND	ND	ND	ND	ND	ND	ND	ND

ND not detected; EHEC: enterohaemorrhagic *E. coli*; EPEC: Enteropathogenic *E. coli*; EIEC: enteroinvasive *E. coli*; EAEC: Enteroaggregative *E. coli*.

## References

[1] MacDonald G.J., Defelice N., Sebastian D., Leker H. (2014). Racial disparities in access to community water supply service in Wake County, North Carolina. Front. Public Health Serv. Syst. Res..

[2] WHO (World Health Organization) (2000). Global Water Supply and Sanitation Assessment 2000 Report.

[3] UN/WHO Clean Water and Sanitation UN-Water Annual Report 2015. www.unwater.org/publications/un-waterannual-report.

[4] Bain R.E., Gundry S.W., Wright J.A., Yang H., Pedley S., Bartram J.K. (2012). Accounting for water quality in monitoring access to safe drinking-water as part of the Millennium Development Goals: Lessons from five countries. Bull. World Health Organ..

[5] WHO (World Health Organization), UNICEF (2014). Progress on Drinking Water and Sanitation: 2014 Update.

[6] WHO (World Health Organization) (2015). Lack of Sanitation for 2.4 Billion People is Undermining Health Improvements. www.who.int.

[7] Safe Water Alliance, Environmental Justice Coalition for Water (2014). Racial Discrimination and Access to Safe, Affordable Water for Communities of Color in California.

[8] Safe Water Alliance, Environmental Justice Coalition for Water (2014). Environmental Justice Coalition for Water, and the International Human Rights Law Clinic.

[9] Department of Water Affairs & Forestry (DWAF) (1994). Water Supply and Sanitation White Paper: Water—An Indivisible National Asset.

[10] Republic of South Africa (1996). Constitution of the Republic of South Africa, 1996 (Act No. 108 of 1996). Gov. Gaz..

[11] Schwartz J., Levin R., Goldstein R. (2000). Drinking water turbidity and gastrointestinal illness in the elderly of Philadelphia. J. Epidemiol. Community Health.

[12] Momba M.N.B., Kaleni P. (2002). Regrowth and survival of indicator microorganisms on the surfaces of household containers used for the storage of drinking water in rural communities of South Africa. Water Res..

[13] Obi C.L., Bessong P.O. (2002). Diarrhoegenic bacterial pathogens in HIV-positive patients with diarrhoea in rural communities of Limpopo Province, South Africa. J. Health Popul. Nutr..

[14] Mackintosh G., Colvin C. (2003). Failure of rural schemes in South Africa to provide potable water. Environ. Geol..

[15] Momba M.N.B., Tyafa Z., Makala N. (2004). Rural water treatment plants fail to provide potable water to their consumers: Alice water treatment plant in the Eastern Cape Province of South Africa. S. Afr. J. Sci..

[16] Obi C.L., Onabolu B., Momba M.N.B., Igumbor J.O., Ramalivahna J., Bessong P.O., Van Rensburg E.J., Lukoto M., Green E., Mulaudzi T.B. (2006). The interesting cross-paths of HIV/AIDS and water in Southern Africa with special reference to South Africa. Water SA.

[17] Momba M.N.B., Madoroba E., Obi C.L., Mendez-Vilas A. (2010). Apparent impact of enteric pathogens in drinking water and implications for the relentless saga of HIV/AIDS in South Africa. Current Research, Technology, and Education Topics in Applied Microbiology and Microbial Biotechnology.

[18] SANS 241 (2006). South African National Standard: Drinking Water Specification.

[19] WHO (World Health Organization), UNICEF (2015). Progress on Sanitation and Drinking Water: 2015 Update and MDG Assessment.

[20] Socio-Economic Profile_Ugu-District 2010. http://www.ugu.gov.za/pdfs/UGU_DM_INVEST_PROFILE.pdf.

[21] Pedley S., Howard G. (1997). The public health implications of microbiological contamination of groundwater. Q. J. Eng. Geol..

[22] Stewart J.R., Gast R.J., Fujioka R.S., Solo-Gabriele H.M., Meschke J.S., Amaral-Zettler L.A., Del Castillo E., Polz M.F., Collier T.K., Strom M.S. (2008). The coastal environment and human health: Microbial indicators, pathogens, sentinels and reservoirs. Environ. Health.

[23] APHA (1992). Standard Methods for the Examination of Water and Wastewater.

[24] EPA (United States Environmental Protection Agency) (2004). Implementation Guidance for Ambient Water Quality for Bacteria.

[25] APHA (2001). Standard Methods for the Examination of Water and Wastewater.

[26] APHA (2005). Standard Methods for the Examination of Water and Wastewater.

[27] Department of Water Affairs and Forestry (DWAF) (1996). South African Water Quality Guidelines for Domestic Use.

[28] Prescott L.M., Harley J.P., Klein D.A. (1993). Microbiology.

[29] Dombek P.E., Johnson L.K., Zimmerley S.T., Sadowsky M.J. (2000). Use of repetitive DNA sequences and the PCR to differentiate Escherichia coli isolates from human and animal sources. Appl. Environ. Microbiol..

[30] Ishii S., Ksoll W.B., Hicks R.E., Sadowsky M.J. (2006). Presence and growth of naturalized *Escherichia coli* in temperate soils from Lake Superior watersheds. Appl. Environ. Microbiol..

[31] Oswald W.E., Lescano A.G., Bern C., Calderon M.M., Cabrera L., Gilman R.H. (2007). Faecal contamination of drinking water within peri-urban households, Lima, Peru. J. Trop. Med. Hyg..

[32] Yamasaki S., Lin Z., Shirai H., Terai A., Oku Y., Ito H., Ohmura M., Karasawa T., Tsukamoto T., Kurazono H. (1996). Typing of verotoxin by DNA colony hybridisation with poly- and oligonucleotide probe, a bead enzyme chain reaction. J. Microbiol. Immunol..

[33] Sethabutr O., Venkatesan M., Marphy G.S., Eampokalap B., Hoge C.W., Echeverria P. (1993). Detection of *Shigella* and enteroinvasive *E. coli* by amplification of the invasion plasmid antigen H DNA sequence in patients with dysentery. J. Infect. Dis..

[34] Ratchtrachenchai O.A., Subpasu S., Ito K. (1997). Investigation on enteroaggregative *Escherichia coli* infection by multiplex PCR. Bull. Dept. Med. Sci..

[35] Mpehle Z. (2015). Socio-economic and spatial inequalities in the provisioning of sustainable housing in South Africa. Politeia.

[36] Ellaway A., Macintyre S., Bonnefoy X. (2005). Graffiti, Greenery, and Obesity in adults: Secondary analysis of European cross Sectional survey. Br. Med. J..

[37] Baker M., Mcnicholas A., Garrett N., Jones N., Stewart J., Koberstein V., Lennon D. (2000). Household crowding a major risk factor for epidemic meningococcal disease in Auckland children. Pediatr. Infect. Dis. J..

[38] Jazairy I., Alamgir M., Panuccio T. (1992). The State of World Rural Poverty: An Inquiry into Its Causes and Consequences.

[39] Sahn D.E., Stifel D.C. (2003). Urban-rural inequality in living standards in Africa. J. Afr. Econ..

[40] Wilson S.M., Heaney C.D., Cooper J., Wilson O. (2008). Built environment issues in unserved and underserved African-American neighborhoods in North Carolina. Environ. Justice.

[41] Wilson S.M., Heaney C.D., Wilson O. (2010). Governance structures and the lack of basic amenities: Can community engagement be effectively used to address environmental injustice in underserved black communities?. Environ. Justice.

[42] WHO (World Health Organization) (2009). Women and Health: Today’s Evidence Tomorrow’s Agenda.

[43] WHO/UNICEF 2016 (2016). Joint Monitoring Programme for Water Supply, Sanitation and Hygiene.

[44] PieTrucha-urbaniK K., Studziński A. (2017). Case study of failure simulation of pipelines conducted in chosen water supply system. Eksploatacja i Niezawodność.

[45] Sorenson S.B., Morssink C., Campos P.A. (2011). Safe access to safe water in low-income countries: Water fetching in current times. Soc. Sci. Med..

[46] House S., Ferron S., Sommer M., Cavill S. (2014). Violence, Gender and WASH: A Practitioner’s Toolkit—Making Water, Sanitation and Hygiene Safer Through Improved Programming and Services.

[47] Firestone L., Kaswan A., Meraz S. (2006). Environmental justice: Access to clean drinking water. Hastings Law J..

[48] Heaney C.D., Wing S., Wilson S., Campbell R., Cadwell D., Hopkins B., O’shea S., Yeatts K. (2013). Public infrastructure disparities and the microbiological and chemical safety of drinking and surface water supplies in a community bordering a landfill. J. Environ. Health.

[49] Doyle J.T., Kindness L., Bear D.W., Realbird J., Eggers M.J. Addressing disparities in safe drinking water access on the Crow Reservation, Montana. Proceedings of the Environmental Health Disparities and Environmental Justice Meeting.

[50] Islam M.S., Hossain M.A., Khan S.I., Khan M.N.H., Sack R.B., Albert M.J., Huq A., Colwell R.R. (2001). Survival of Shigella dysenteriae Type 1 on fomites. J. Health Popul. Nutr..

[51] Trevett A.F., Carter R.C., Tyrrel S.F. (2005). The importance of domestic water quality management in the context of faecal-oral disease transmission. J. Water Health.

[52] Peter G. (2010). Impact of rural water projects on hygienic behaviour in Swaziland. Phys. Chem. Earth.

[53] Blum D., Emeh R.N., Huttly S.R.A., Dosunmu-Ogunbi O., Okeke N., Ajala M., Okoro J.I., Akujobi C., Kirkwood B.R., Feachem R.G. (1990). The Imo State (Nigeria) drinking water supply and sanitation project, 1. Description of the project, evaluation methods, and impact on intervening variables. Trans. R. Soc. Trop. Med. Hyg..

[54] Singh U., Lutchmanariyan R., Wright J., Knight S., Jackson S., Langmark J., Voslooand D., Rodda N. (2013). Microbial quality of drinking water from ground tanks and tankers at source and point-of-use in eThekwini Municipality, South Africa, and its relationship to health outcomes. Water SA.

[55] Trevett A.F., Carter R.C., Tyrrel S.F. (2004). Water quality deterioration: A study of household drinking-water quality in rural Honduras. Int. J. Environ. Health.

[56] Clasen T., Roberts I., Rabie T., Schmidt W., Cairncross S. (2005). Interventions to Improve Water Quality for Preventing Infectious Diarrhoea.

[57] Yang Z., Wu X., Li T., Li M., Zhong Y., Liu Y., Deng Z., Di B., Huang C., Liang H. (2011). Epidemiological survey and analysis on an outbreak of gastroenteristis due to water contamination. Biomed. Environ. Sci..

[58] Block J.C., Haudidier K., Paquin J.L., Miazga J., Levi Y. (1993). Biofilm accumulation in drinking water distribution systems. Biofouling.

[59] Camper A.K., Geesey G.G., Lewandowski Z., Flemming H.-C. (1994). Coliform regrowth and biofilm accumulation in drinking water systems: A review. Biofouling and Biocorrosion in Industrial Water Systems.

[60] Costerton J.W., Lewandowski Z., De Beer D., Caldwell D., Korber D., James G. (1994). Biofilms, the customised microniche. J. Bacteriol..

[61] WHO (World Health Organization) (2008). Guidelines for Drinking-Water Quality.

[62] Department of Water Affairs & Forestry (DWAF) (1996). South African Water Quality Guidelines—Volume 1: Domestic Water Use.

